# Prospective Lymphedema Surveillance in a Clinic Setting

**DOI:** 10.3390/jpm5030311

**Published:** 2015-08-25

**Authors:** Janet Chance-Hetzler, Jane Armer, Maggie Van Loo, Blake Anderson, Robin Harris, Rebecca Ewing, Bob Stewart

**Affiliations:** 1 Sinclair School of Nursing, University of Missouri-Columbia, S235 School of Nursing Building, Columbia, MO 65211, USA; E-Mails: chancehetzlerj@missouri.edu (J.C.-H.); blake@expressiveanalytics.com (B.A.); harrisrc@missouri.edu (R.H.); stewartb@missouri.edu (B.S.); 2 Lymphedema Research Laboratory, Sinclair School of Nursing, University of Missouri, DC 116.05, Suite 408, Mizzou North Campus, Columbia, MO 65211, USA; 3 Ellis Fischel Cancer Center, One Hospital Drive, Columbia, MO 65212, USA; E-Mail: vanloomj@health.missouri.edu; 4 Statistics Department, University of Missouri-Columbia, 23 Middlebush Hall, Columbia, MO 65211, USA; E-Mail: rje88b@missouri.edu

**Keywords:** lymphedema, surveillance, cost, referral

## Abstract

The potential impact of breast cancer-related lymphedema (LE) is quite extensive, yet it often remains under-diagnosed until the later stages. This project examines the effectiveness of prospective surveillance in post-surgical breast cancer patients. A retrospective analysis of 49 out of 100 patients enrolled in a longitudinal prospective study at a Midwestern breast center evaluates: (1) time required for completion of bilateral limb measurements and Lymphedema Breast Cancer Questionnaire (LBCQ); (2) referral to LE management with limb volume increase (LVI) and/or LBCQ symptoms; and (3) cost of LE management at lower LVI (≥5%–≤10%) versus traditional (≥10%). Findings revealed a visit timeframe mean of 40.3 min (range = 25–60); 43.6% of visits were ≤30-min timeframe. Visit and measurement times decreased as clinic staff gained measurement experience; measurement time mean was 17.9 min (range = 16.9–18.9). LBCQ symptoms and LVI were significantly (*p* < 0.001) correlated to LE referral; six of the nine patients referred (67%) displayed both LBCQ symptoms/LVI. Visits with no symptoms reported did not result in referral, demonstrating the importance of using both indicators when assessing early LE. Lower threshold referral provides compelling evidence of potential cost savings over traditional threshold referral with reported costs of: $3755.00 and $6353.00, respectively (40.9% savings).

## 1. Introduction

The American Cancer Society [1] estimates that there are more than 2.9 million women living with breast cancer in the United States and annually approximately 235,000 women will develop breast cancer. It is further approximated that of these two million plus breast cancer survivors, one-third to one-half will develop lymphedema (LE) during the course of their lifetime [2]. With the improving breast cancer survival rate [1] and survivors being at a life-time risk for developing LE, there is a growing population of women at risk for the development of this complication [2,3], particularly those patients who have undergone lymph node dissection and/or regional radiation [4]. While the potential impact of LE is quite extensive, it remains largely unrecognized, under-diagnosed, and untreated until the later more visible stages [3]. When LE is detected in earlier stages, therapeutic management is more likely to be effective in improving outcomes and quality of life [3].

### 1.1. Background and Significance

LE is defined as an accumulation of high-protein concentrated fluid in the interstitial spaces [4]. Classified as either primary or secondary (acquired), it is caused by a disruption or malformation of the lymphatic system [5]. Primary LE has no known cause and develops from an insufficiency in the structure or function of the lymphatic system [4] and can be congenital, developing at the onset of puberty, or in adulthood potentially affecting all limbs and parts of the body [5]. Acquired or secondary LE is the more common, with surgical or radiation therapy for breast cancer being the most common cause [4]. Secondary LE is arguably the most problematic and dreaded complication of breast cancer treatment [6]. Post breast cancer-related LE (BCRL) usually results after mastectomy or lumpectomy with axillary node dissection or sentinel node biopsy (although the latter is less prevalent), radiation therapy, or other trauma to the area [7]. Onset may be gradual or sudden, with 15%–54% developing LE within three years of their breast cancer diagnosis [8], although, it can appear much later (as much as 30 years post-diagnosis), placing women at a lifetime risk for post-treatment LE development [9]. A study conducted by Boccardo *et al.* [10] reported approximately 75% of LE cases occur in the first year after the breast cancer surgical intervention. Once LE manifests itself, it is considered to be a chronic and life-long condition [11], due to the permanent damage to various lymphatic components [12]. Without intervention, LE can lead to progressive swelling, fibrosis of the soft tissues, neurologic changes (such as pain and/or paresthesias), and infection [13]. This resulting arm morbidity can cause alterations in function with ensuing adverse physical and psychosocial ramifications that can profoundly affect the quality of life (QOL) in breast cancer survivors [13]. The psychosocial impact of a LE diagnosis has been found to be as distressing to breast cancer survivors as their initial diagnosis of breast cancer [14]. Early identification of the signs and symptoms of LE can be integral to the management of all patients who have received surgery and/or radiation, and are thus at high risk for the development of LE [15]. When treated in the earliest stages, the more severe complications of LE may be minimized, which will improve QOL and functional outcomes [12].

In an effort to effectuate earlier identification and treatment of LE, it is recommended that patients be screened for low-level arm volume changes to enable earlier referral and intervention [2,10,16]. The most common diagnostic criteria used for LE is a difference of 200 mL or 10% difference limb volume increase (LVI) or a 2 cm difference in contralateral limb circumference [2,10]. A study conducted by Cormier *et al.* [17] found a 5.0% LVI to be associated with increased symptoms of swelling and heaviness and a decreased QOL. Unfortunately, most lymphedema is not diagnosed until the later stages, when it becomes visibly apparent. A study conducted by Specht *et al.* [18] suggests that a lower threshold of a ≥5 to <10% relative volume change (RVC) criterion be utilized for referral for further assessment or intervention, as crossing this threshold was a significant predictor of progression to ≥10% RVC. While treatment can begin at any stage of LE, outcomes are less optimal in the later stages due to the adipose and fibrotic changes within the tissues [18]. Therefore, prospective surveillance plays an important role in the earlier detection and management of LE.

## 2. Review of Literature

A search was conducted utilizing the National Library of Medicine (MEDLINE), Cumulative Index to Nursing & Allied Health Literature (CINAHL), PubMed, Scopus, and Ovid databases. These databases were searched using the search terms, “breast cancer”, “lymphedema screening”, “diagnostic tools”, “breast cancer follow-up care“, “Lymphedema Breast Cancer Questionnaire”, “lymphedema”, “survivor”, long-term”, “early intervention”, “cost effectiveness”, “threshold for intervention”, “late effects”, and “post-surgical follow-up”. Studies included were conducted and published in the United States and Canada between 2003 and 2014.

### 2.1. Prospective Surveillance Model

Historically, water displacement volumetry was the “gold standard” of measurement for clinical evaluation of patients presenting with limb swelling since it offers a sensitive and accurate volume measurement [13]. Perometry is also another tool used to measure and evaluate LE, but perometer and water displacement may not be perceived as conducive in all clinic settings due to space limitations and ease of use [13]. Thus, the optimal method for obtaining limb measurements may differ between institutions. Currently, most clinical settings use serial circumferential limb measurements to assess for LE [19]. Several studies have suggested that subjective assessment through patient self-reported changes (e.g., limb heaviness, swelling, change in fit of garments, redness, and tenderness) and functional changes (e.g., reduced range of motion) is sensitive to the development of LE [2,3,4,7,8,12]. In addition to being less expensive, it also allows for detection at an earlier stage of development [3]. A combination of symptom assessment and circumferential limb measurement may provide a more comprehensive assessment of early limb changes, which is more conducive for use in the clinic setting [3,20,21].

Prospective monitoring for early identification of BCRL is not standard practice in the clinic setting, and even when issues are identified, access and referral to prompt, appropriate therapy services may be lacking [22]. Singh, De Vera, and Campbell [22] conducted a pilot study comparing the effect of arm morbidity in post-surgical breast cancer patients involved in a clinical care pathway, inclusive of preoperative education, prospective monitoring, and early physiotherapy (experimental group) to perioperative education alone. Findings from this study reported persistent arm morbidity at 7-months follow-up in 32% of the women enrolled. This study also reported a lower incidence of arm morbidity and better QOL in the experimental group, with the majority of women identified to receive physiotherapy only requiring one or two additional visits to address the issue. However, due to the quasi-experimental design, the participants were not randomized to the experimental group. Therefore, there may have been differences in participant characteristics and surgical approaches resulting in an unequal risk for development of arm morbidity. While these findings were not statistically significant, the results of the study did provide support for integration of surveillance and an early physiotherapy approach to follow-up care in the post-surgical breast cancer setting.

Stout-Gergich *et al.* [23] found that pre-operative LV measurement, combined with post-operative follow-up to identify and treat sub-clinical LE with compression, was associated with LVs returning to normal values. The mean effect LVI reported was 83mL at onset (*p* = 0.005) compared to baseline. After compression intervention, a mean 48mL volume decrease was realized (*p* < 0001). The mean duration of the intervention was 4.4 weeks (±2.9 weeks), and volume reduction was maintained an average of 4.8 months (±4.1 months) post intervention. The trial incorporated a case-control design, which did not control for breast cancer treatment side effects that may have contributed to LE onset and the outcomes reported with the compressions therapy. Due to the study design, the LV changes may have been post-operative swelling, which could have decreased without the use of compressive therapy. However, the findings from this study do support a shift from the current impairment-based treatment paradigm to early intervention.

In a literature review conducted by Binkley *et al.* [24], the authors present the patient perspective for a prospective surveillance model (PSM) in the breast cancer follow-up setting. Binkley *et al.* [24] report that many women find the threat of LE more distressful than their breast cancer. They also found that few breast cancer patients are referred to rehabilitative services and even fewer receive baseline assessments to facilitate early detection of BCRL. These findings are consistent with those reported in a cross-sectional study conducted by Cheville *et al.* [25] which reported 92% of women with metastatic breast cancer had physical impairments related to arm morbidity, yet less than 30% had received referral and treatment.

#### 2.1.1. Lymphedema Breast Cancer Questionnaire Tool

The LBCQ is a structured, self-report tool designed to assess indicators of LE and their frequency. The LBCQ assesses 19 symptoms occurring now or in the past year. The reliability of the LBCQ was evaluated by using Kuder-Richardson-20 and the test-retest method. The Kuder-Richardson-20 shows an acceptable measure for internal consistency (*r* = 0.785); test-retest showed a high degree of reliability (*r* = 0.98) [26].

A study completed by Armer, Radina, Porock, and Culbertson [3] evaluated the validity and accuracy of using the LBCQ self-report tool to differentiate between women with and without LE. In study A, logistic regression was used to elucidate symptoms predictive of LE between participants known to have LE (*n* = 40) and a control group of women with no history of breast cancer or LE. Symptoms experienced in study A were then used in a second independent data set (*n* = 103) in which a diagnosis of LE had not been previously determined. Findings from study A suggest changes in sensation and range of motion might be an early indicator of LE development and/or other treatment-related sequelae. Two symptoms, heaviness in the past year (*p* = 0.0279) and swelling now (*p* = 0.0007), were found to be significant in identifying those women with LE. These symptoms were highly reported in women with LE, versus rarely by women without LE. Subsequent studies have also supported subjective assessment through patient self-report as being sensitive to the development of LE, as well as substantially less expensive than other forms of monitoring [2,27].

#### 2.1.2. Limb Measurements

Lawenda, Mondry, and Johnstone [12] identify the preoperative evaluation as being a key component to the early detection and treatment of LE. The findings from this review present early identification of the signs and symptoms of LE as being integral to the management of all breast cancer patients who receive surgery and/or radiation therapy. These authors also support obtaining baseline (pre-operative) limb measurements of girth and volume which can assist in finding any subsequent changes in the size of the limb during follow-up appointments, allowing for earlier intervention.

A study conducted by Armer *et al.* [3] reported findings that suggest that changes in sensations may be early indicators of LE that should be assessed at each follow-up visit. Armer *et al.* [3] state that a combined limb measurement and symptom assessment may provide the most accurate data for identifying changes associated with BCRL and support an approach of using patient self-report and objective measure by obtaining physical anthropometric measures (*i.e.*, comparison of circumferential measurement of upper extremities). In this study LE prediction was based upon a >2 cm difference in circumferential measurements.

#### 2.1.3. Cost

The rationale for implementation of prospective LE surveillance is the potential to reduce the severity of physical impairments experienced by breast cancer survivors. There is very little literature on the economic impact of BCRL and poorly managed BCRL may lead to complications needing more costly medical interventions, which may increase cost of care. Another rationale for a PSM for BCRL is the presumption that an earlier identification and treatment of BCRL will lessen future morbidity and health-related issues, which would in turn reduce the costs of treatment.

A Canadian study conducted by Keast, Allen, Despatis, and Brassard [28] reported BCRL not identified in the earlier stages to have a much poorer prognosis and significantly higher costs of treatment. Their findings suggest that the disparities between later diagnosis and treatment of LE create a poorer prognosis for the breast cancer survivor, with more costly, less effective treatments as compared to LE identified and treated in the earlier stages.

In addition, Stout *et al.* [23] report significantly increased costs associated with later-stage diagnosis in the prospective cost analysis they conducted, which compared a PSM for BCRL with a traditional model of impairment-based care and examined the direct treatment costs associated with each program. The PSM included the cost of screening all the women enrolled plus the cost of intervention for early-stage BCRL. The traditional model included women referred for BCRL treatment who were in late-stage LE, representing the direct costs of treating patients with advanced-stage LE. The authors reported the per patient cost to manage early-stage BCRL using a PSM is $636.19, as compared to $3,124.92 per patient cost to manage late-stage BCRL. The findings of the study suggest that a comprehensive PSM of care is not only optimal for early detection of BCRL, but may provide substantial cost savings over an impaired-based care approach to BCRL. Limitations of the findings of this study are associated with the use of the Medicare fee schedule for analysis, which may underestimate the true cost of treatment of LE; the study also is a prospective cost analysis so data for direct and indirect costs of PSM are lacking.

A study conducted by Shih *et al.* [29] similarly reported that BCRL increased the 2-year, post-operative medical costs by $14,877–$23,167. These additional costs were attributed to more office visits; diagnostic imaging of upper arm, chest and abdomen; treatments for the development of infections; and mental health services, inclusive of prescriptions for antidepressants. This study also found that women with BCRL suffer greater loss of productive days than those without LE (73 versus 56 days).

A prevalence study conducted by Moffatt *et al.* [30] reported that for every $1 spent on LE treatment, there resulted a $100 cost savings in hospital admissions. These findings support early intervention and management of LE as key factors for reducing unnecessary, avoidable costs to the United Kingdom health care system. This study found that 29% had an infection in the past 12 months; 27% of patients with LE were admitted to hospitals for antibiotic treatment, with a mean length of stay of 12 days; 32% received some form of compression therapy; 80% had required time off from work; and 8% of patients had to give up work.

The major aim of this project is to determine the feasibility of incorporating a PSM in routine post-surgical breast cancer clinic follow-up assessment using anthropometric limb measurement and symptom assessment with the LBCQ tool. A second aim of this project was to evaluate identification and referral of post-surgical breast cancer patients, with a 5% increase in LV over baseline or with patient reports of arm feeling heavy or swollen as assessed by the LBCQ tool, to rehabilitative services for further evaluation. The final aim of this project was to evaluate the cost of treatment in patients identified and referred in the early stage versus the later stage of LE.

## 3. Results

This project represents a retrospective, descriptive secondary analysis of patients enrolled in a longitudinal prospective LE Surveillance study at a nationally-recognized, university-affiliated breast center in the Midwest. Data were extracted from 49 patient research charts, electronic medical records, and the institutional billing records in an attempt to determine the effectiveness and cost feasibility of implementation of a PSM for early identification of LE in a post-surgical breast cancer clinic follow-up setting. The median age of the enrolled sample was 59 years old, with a range of 34–81 years. The ethnicity of the sample was predominantly Caucasian (47/49; 95.9%), with only 4.1% (2/49) self-identifying as African American.

### 3.1. Clinic Time

**Figure 1:**
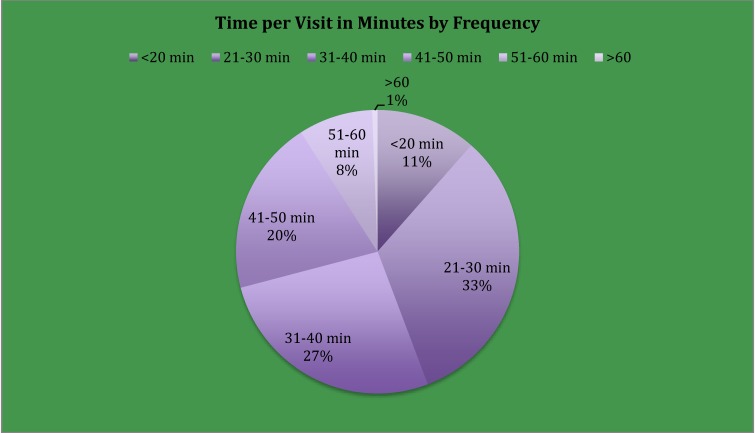
Time per Visit in Minutes by Frequency.

**Figure 2:**
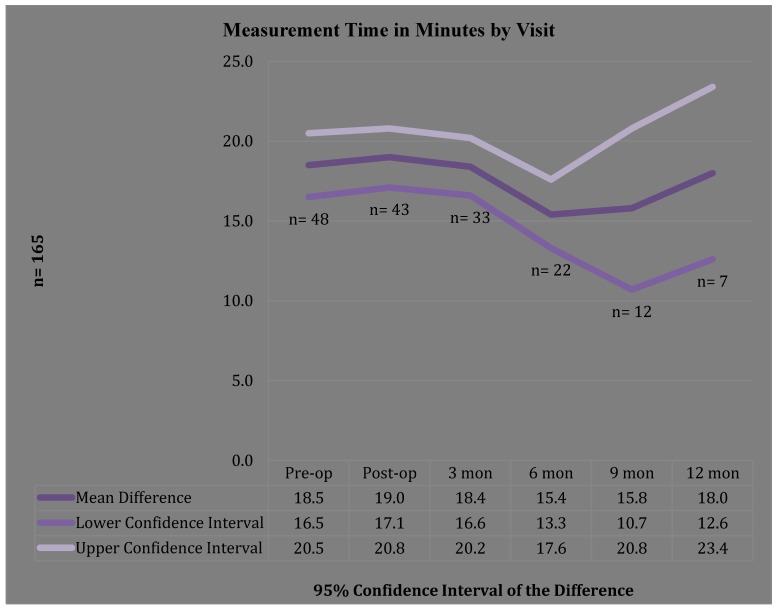
Measurement Time in Minutes by Visit.

**Figure 3:**
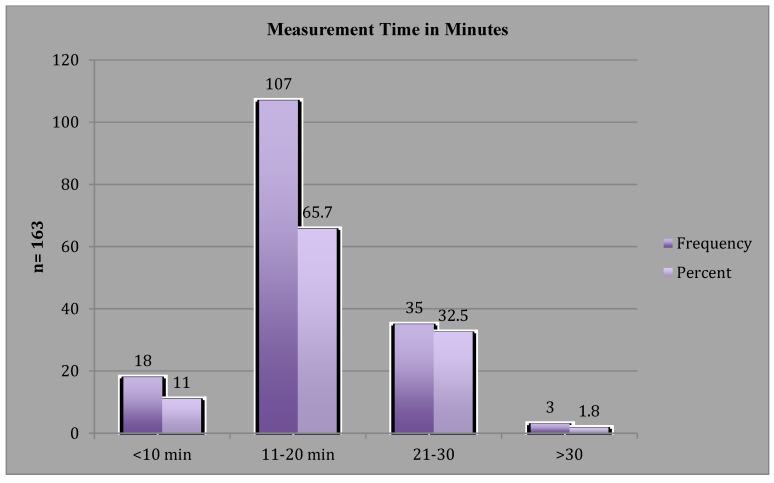
Measurement Time in Minutes.

Visit frequency (*n* = 165) data revealed that 72/165 visits (43.6%) were completed in 30 min or less, while 93/165 (56%) exceeded 30-min timeframe. However, time per visit in minutes by frequency (Figure 1) found 71% of the visits were completed in ≤ 40 min. A one-sample T-Test of the time per visit (pre-op, post-op, and 3-, 6-, 9-, 12-months) in minutes visit times revealed a significant change in per measurement time (*p* = 0.000) with visit time decreasing as number of participants increased and staff experience with assessment tools increased (Figure 2). The time required for circumferential measurement (Figure 3) in minutes by frequency (*n* = 163) revealed the majority of measurements were completed in 20 min or less (76.7%), with a one-sample T-Test revealing a mean of 17.9 min (range = 17–19) with a significant change in per measurement time (*p* = 0.000). Findings revealed a visit timeframe mean of 40.3 min (range = 25–60), 43.6% of visits were ≤30-min timeframe. Measurement time in minutes by visit revealed the post-op visit time increased over other visit times (Figure 2).

### 3.2. Referral to LE Management

Referral to rehabilitative services for further evaluation was significantly correlated (*p* < 0.001) with the presence of both LBCQ symptoms and LVI (Table 1). Patients presenting with LBCQ symptoms only (*n* = 17) or LVI only (*n* = 13) were much less likely to be referred to rehabilitative LE treatment (5.9%, 15.4%, respectively). In patient visits with no reported symptoms, 85/115 (73.9%) resulted in no referrals (100%).

**Table 1:** LBCQ Symptoms/LVI Referral.

Referral * Symptoms Crosstabulation				
Valid Number of responses = 115				
	No Symptoms	LBCQ Sym	LVI = 5%	Both LBCQ/LVI
n	85(73.9%)	17(14.8%)	13(11.3%)	9(7.8%)
Referral No	85	16	11	3
Referral Yes	0	1	2	6
% not Referred	100.0%	94.1%	84.6%	33.3%
% Referred	0%	5.9%	15.4%	67.0%
**Chi-Square Test**				
	**Asymp. Sig**		**Exact Sig**	
Pearson Chi-Square	*p* = 0.000		*p* = 0.000	

### 3.3. Treatment Costs

The LE management costs analysis comparing referral of two patients, reported cost savings of $3755.00 at the reduced threshold of 5% LVI versus $6353.00 for referral point at ≥10% ≥LVI, for a cost savings of 40.9%. The LVI experienced in the surveillance patient returned to pre-op levels, with no ongoing charges, while the comparison patient referred at the traditional LVI continues to receive treatment with ongoing charges.

## 4. Discussion

The findings of this study reveal that while the comprehensive visit times (inclusive of subjective/objective assessments) were not completed within the routine 30-min appointment timeframe, the majority exceeded this time by only a few minutes, suggesting that as staff and participants gain experience with the surveillance process, time required would decrease. This was further evidenced when time required to obtain the circumferential measurements were analyzed separately, revealing that the majority of measurements were completed in ≤20 min. Measurement time in minutes by visit revealed the post-op visit time increased over other visit times, which the measuring staff attribute to the post-operative discomfort experienced by the patients, which required additional time for positioning during measurements. The pre-op per measurement mean was 18.50 min, the mean increases to 19 at post-op; the three-month mean decreased to 18.4 min; the six-month mean further decreased to 15.4 min; the mean shows an increase to 15.8 min at 9 months and 18 min at 12 months, which perhaps be explained by the decreasing sample size for these measures, *n* = 12 and *n* = 7, respectively.

It is also important to note the pre-op and 12-month visit times were inclusive of the completion of the FACT B + 4 (HRQOL) survey; the 12-month visit time also included a patient satisfaction survey.

Referral to an LE specialist for evaluation was significantly correlated with the presence of both subjective and objective assessment findings. Patients presenting with independent subjective or objective symptoms were much less likely to be referred for evaluation. Patient visits with no reported symptoms (subjective/objective) were not referred for evaluation, demonstrating the tool’s ability to differentiate between those patients with symptoms and those without. The potential effect of size differential between limbs (dominant/non-dominant) and changes in body mass were controlled by obtaining a preoperative measurement as a baseline and serial measurements. These collective findings suggest that the utilization of objective and subjective indicators in prospective surveillance for LE assessment may be more effective than using a single diagnostic method.

While it would be presumptuous to make the claim that prospective surveillance positively impacted the cost of LE management, the cost analysis comparing referral at the reduced threshold versus the traditional referral point did provide compelling evidence of cost savings (40.9%.). While there have been studies that have shown the projected cost estimates of LE management [10,16], there have been no studies analyzing actual costs of LE management. The 2011 National Lymphedema Network position statement highlights PSM as a standard of care for early identification of LE [31]; yet PSM is not common in the outpatient clinical setting. The findings of this study also suggest PSM as potentially decreasing treatment costs for managing BCRL.

### Limitations

We believe that our findings offer promising evidence of the feasibility of a PSM for early LE identification and management. However, this study had several limitations, such as the small sample size and the inability of the researcher to capture charges for participants who were referred for evaluation outside of the local health care system. Another potential limitation to the study was the referral point to LE evaluation being any measurement of LVI ≥5% and <10%, which could have captured transient edema that would have potentially resolved without intervention. The study also likely underestimated the cost of LE management, as this study used International Classification of Diseases-codes in claims data specific to LE and did not evaluate other associated ancillary charges. Obtaining circumferential measurements can be time-consuming and control of intra- and inter-rater reliability is difficult. Another limitation of this study is that currently level 1 evidence does not exist to support intervention for LE evaluation at 5%–10% LVI and access to LE specialists can be limited, potentially resulting in long wait times.

## 5. Materials and Methods

This is the original study for these data; the data and protocols from an NIH-funded longitudinal prospective surveillance research study were applied to the clinical setting for this feasibility study. Upon receiving Institutional Review Board approval, the parent study consecutively recruited and enrolled patients at a nationally recognized, university-affiliated breast center after obtaining informed consent. In this project a retrospective medical record review was conducted to evaluate:

- Sequential bilateral circumferential limb measurements, obtained during clinic visits preoperatively, and at 3-, 6-, 9-, 12-months postoperatively;
- Self-report symptoms assessments, obtained using the LBCQ tool by interview or patient entry into the web-based survey, obtained during clinic visits preoperatively, and at 3-, 6-, 9-, 12-months postoperatively, and FACT B+4 quality of life questionnaire, obtained preoperatively and 12-months postoperatively, and participant satisfaction with electronic capture of symptoms obtained at 12-months postoperatively;
- Referral point to LE specialist for assessment in patients presenting with a 5% LVI over baseline and/or reports of arm ‘feeling heavy’ in the past year, or ‘swelling now’ per the LBCQ tool;
- Nursing time required for the assessment (assessment start and stop times), inclusive of completion of LBCQ tool and circumferential measurements; and
- Institutional billed charges; comparisons were analyzed between a patient referred for treatment at the LE Surveillance parameter of ≥5% and <10% LVI versus a patient referred per the traditional parameter of ≥10% LVI.

Extracted data were collected, coded, and entered into an Excel spreadsheet; SPSS version 22 [32] was used to perform descriptive and inferential analyses. This analysis was conducted using frequency; confidence intervals and one-sample T test for visit times and measurement time calculations. Chi-square was used to test the independence of the two categorical variables of LVI and LBCQ symptoms and correlation to referral to rehabilitative services. The cost analysis was undertaken from an institutional perspective using only direct treatment cost associated with LE. Interventions using the following diagnostic codes: 457.0 post-mastectomy lymphedema, 457.1 other lymphedema changes. Direct treatment costs were defined as the cost of the treatment visit with the physical therapist, the cost of medical equipment associated with the condition treatment and management. Forty-nine patient charts were reviewed, nine of these patients were lost to attrition: one due to not meeting study criteria (no data were collected), two were deceased, six dropped out of study (three due to medical reasons and three due to time constraints).

### Limb Measurements

The measurement staff consisted of an advanced practice nurse and two licensed practical nurses; they were trained over several months prior to the clinical trials initiation to perform the circumferential measurements. From the beginning of training and throughout this study, inter and inter-rater reliability measurements were obtained monthly with a model patient measured by the research team. Measurements have maintained high reliability throughout the study. Estimated standard deviations of within-nurse and between-nurse reliability measurements have consistently been in the 0.25 to 0.35 cm range and in the 0.10 to 0.20 cm range, respectively. Circumferential measurements were conducted using a flexible tape (to assure appropriate tension is achieved over soft tissue and bony prominences). Measurements were obtained in the affected and unaffected arm, beginning with the hand (proximal to the metacarpals) and wrist, and up the limb from the wrist to the axilla in 4 cm increments, with three consecutive measurements of each limb to ensure accuracy.

## 6. Inclusion Criteria

The sample enrolled in the study were limited to: Women age 18 years or older; newly diagnosed with Stage I-IV cancer of the breast; who may have received any type of surgery or radiation therapy to the breast or axilla; had no prior diagnosis of LE; were not currently homebound or required the use of a walker/wheelchair for mobility and willing to return to the study site for the duration of treatment follow-up (12 months).

## 7. Conclusions

When LE is detected at its earliest onset, therapeutic management has a greater likelihood of reducing short- and long-term morbidity. Assessing subjective, as well as objective, signs and symptoms through a PSM approach during routine follow-up appointments holds the greatest promise for earlier identification and treatment of LE. Prospective surveillance with early educational and preventative measures may translate to more treatment options; improved treatment costs, and an improved QOL in patients affected by LE.

## 8. Future Directions

Recommendations for future research include a larger sample size with inclusion of the surgical approach and nodal status to include in the statistical analysis. Future research is also needed to evaluate the long-term clinical benefits and cost-effectiveness of a PSM for LE, as compared to the current impairment-based treatment model.
